# The effect of double nursing, an alternative nursing strategy for the hyper-prolific sow herd, on herd performance

**DOI:** 10.1186/s40813-016-0050-1

**Published:** 2017-02-16

**Authors:** Manon A. M. Houben, Tijs J. Tobias, Manon M. C. Holstege

**Affiliations:** 1 GD Animal Health Service, PO box 9 7400 AA, Deventer, The Netherlands; 2 Department of Farm Animal Health, Utrecht University, Faculty of Veterinary Medicine, Yalelaan 7, 3584 CL, Utrecht, The Netherlands; 3 PorQ BV, P.O. Box 52, 5690 AB, Son, The Netherlands

**Keywords:** Pig, Sow, Mortality, Farrowing house management, Cross fostering

## Abstract

**Background:**

Hyper-prolific sows produce more piglets than they can suckle, as the number of milk producing teats of the sow is lower (twelve to sixteen) than the number of live born piglets per litter. Farmers and farm workers are struggling to feed this surplus of piglets. To minimize suckling piglet mortality, litter size at 24 hours after parturition should not exceed the number of functional teats of the sow. Strategies to adequately nurse or feed the surplus of piglets after 24 hours are limited and mostly restricted to either fostering piglets by other sows, supplying milk replacers (formula) or early weaning and rearing on formula.

**Case presentation:**

In this case report we describe the design and application of a so called ‘double nursing’ strategy, for which one sow simultaneously nurses two litters from birth to weaning. Piglet mortality and reproductive parameters of sows that have nursed two litters are compared, over a three year period, with those that nursed one litter.

**Conclusion:**

In this herd, the double nursing strategy appeared to be a successful strategy. Double nursing sows experienced a lower piglet mortality, despite the double nursing strategy. The negative effects on reproduction proved to be limited, there was a negative effect on litter size in subsequent litters, but no significant effect on the interval weaning to next conception. It has to be noted though that not all characteristics on which double nursing selection takes place, could be taken into account during statistical analyses.

## Background

Hyper-prolific sows produce more piglets than they can suckle [1]. During the first hours after parturition, piglets can consume colostrum continuously, as it is available independent of the nursings of the sow [2]. Strategies that ensure intake of colostrum by all piglets of hyper prolific sows on farms include split suckling as well as oral supplementation of colostrum [3–6]. Although little information is available about the changes in colostrum and milk availability for the piglets, according to Farmer, colostrum availability will gradually change within the first day post-partum from being available continuously to being available after milk ejections [2, 7]. In the first hours these ejections will occur spontaneously, but in time the ejections are dependent on the tactile stimulation of the udder skin. From approximately 16–24 hours after parturition [8], milk is only available for piglets when sows are stimulated and milk is actively ejected by contraction of the myoepithelial cells of the milk alveoli in the mammary gland. The ejection of milk is synchronized for all teats by endocrine signaling, which means that piglets cannot consume milk during the intervals between milk ejections [2]. When litter size exceeds the number of functional teats, piglets are unable to secure their own teat and risk starvation or runting due to competition and inadequate suckling [9]. To minimize suckling piglet mortality, litter size at 24 hours after parturition should not exceed the number of functional teats of the sow. Strategies to adequately nurse or feed the surplus of piglets after 24 hours are limited and mostly restricted to either fostering piglets by other sows [3–5], supplying milk replacers (formula) or early weaning and rearing on formula [6, 10].

## Case presentation

The herd described in this case is a hyper prolific sow herd of 550 sows, producing over 30 weaned piglets per sow per year. In 2011, the nursing strategy was a cross fostering strategy where some nursing sows nursed two successive litters, during one lactation (foster sows) [6, 11]. However, the main side effect of this strategy within this herd was the early weaning of piglets 1) to create foster sows and 2) to solve stocking problems due to pens occupied by foster sows. This nursing and weaning management resulted in an increased difference in the age of piglets in weaner rooms. Also, this herd is known for recurrent problems with weaning diarrhea due to *Escherichia coli*. So to reduce the age difference of the piglets at weaning and to ensure that all piglets are nursed and protected by lactogenic immunity for four weeks [12, 13], it was decided to implement a different nursing and fostering strategy. A nursing strategy was designed based on the split suckling strategy [4–6], often applied in sow herds in the first day of parturition, combined with the intermitted suckling strategy [14–16] and it was named “double nursing (DN)”. In the double nursing strategy every week one or more sows nurse two litters simultaneously from birth to weaning.

The DN strategy was implemented in 2012. The ultimate goal of DN was: 1) to increase the main weaning age 2) prevent piglets to be weaned before four weeks of age and 3) to reduce the age difference of piglets weaned at the same day. Due to this strategy double nursing sows (DNS), sows that nurse two litters, weaned 21 to 28 piglets per lactation. Feed advisors and the herd veterinarian raised concerns regarding the effect of DN on future sow performance. As in literature this nursing strategy is not described, a retrospective herd performance data analysis was performed, on data from three years, to assess the effect of the double nursing on the piglet mortality in current lactation and reproductive parameters of the subsequent cycle.

## Material and methods

### Double nursing definition

**Fig. 1:**
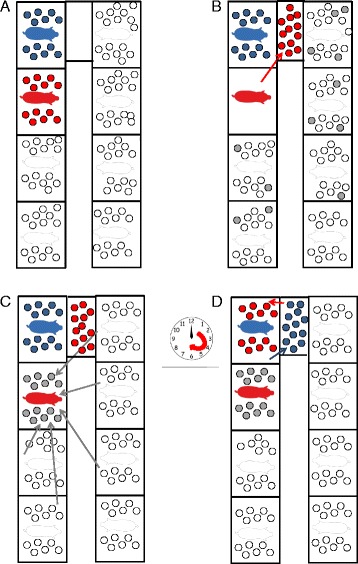
Schematic drawing of the double nursing strategy in a farrowing room. Double nursing sows (DNS) nurse two matched groups of piglets from approximately 12 to 24 hours after parturition until weaning. The two groups are matched by age and number of piglets. **a**) One group consists of the litter born from the DNS (blue) and the other group consists of an entire litter from one other sow (red). **b**) The sow of this litter is then available as foster sow for the surplus piglets (grey) of the same farrowing batch. **c**) One group is housed in the farrowing crate with the DNS, whereas the other group is housed separately in a small pen with a heater, a drinking nipple and milk formula in a feeding through. **d**) Twice a day, preferably every 12 hours, both groups are exchanged

Double nursing sows (DNS) nurse two matched groups of piglets from approximately 12 to 24 hours after parturition until weaning. The two groups are matched by age and number of piglets. One group consists of the litter born from the DNS and the other group consists of an entire litter from one other so called donor sow (Fig. 1). The donor sow is then available as foster sow for the surplus piglets of the same farrowing batch. During lactation the DNS are housed in standard farrowing pens. Double nursing will start after the piglets of both litters have drunk colostrum of their own mother. One group is housed in the farrowing crate with the DNS, whereas the other group is housed separately in a small pen with a heater, a drinking nipple and milk formula in a feeding trough. Twice a day, preferably every 12 hours, both groups are exchanged (Fig. 1).

### Sows

Sows, selected for DN, have to meet the following criteria: 1) parity two or older, 2) a well-developed udder with a minimum of fourteen producing teats, 3) a calm and motherly behavior of the sow (i.e. the sow is attentive and caring for her piglets. These sows interact positively with their piglets. When nursing she will present her udder well and when lying down she does this carefully and slowly), 4) no dystocia or feed intake problems around farrowing and 5) uniformly sized piglets of her own litter. All sows within a weekly batch of farrowing sows are assessed by the farmer and sows that fulfill these criteria after farrowing are immediately selected for DN. The additional double nursing litter is selected by the farmer from other sows. This donor sow should also express 1) a motherly behavior, 2) have not experienced problems around parturition and 3) her piglets should match the piglets of the DNS. This additional litter needs to match the age of the other litter and preferably the weight of the piglets is similar.

### Herd

DN was applied in a multiplier sow herd of 550 sows with a weekly farrowing of 25 sows. The sows were a Landrace x Large White crossbred (Topigs 20) and gilts were introduced to the breeding stock at 6 months of age. Gestating sows were group-housed in one dynamic sow group with straw bedding and electronic feeding stations from 4 days after insemination until one week before parturition. In the farrowing compartment, sows were housed individually in farrowing crates. Farrowing pens were equipped with a so called balance frame (by Nooyen Pig Flooring, Deurne, The Netherlands), which moves sows up when the sow is standing. The double nursing strategy for the surplus of piglets was implemented in this herd in 2012 and is currently still being applied.

### Technical performance data

To evaluate the effects of DN on performance of current (=defined by nursing) litter and subsequent litter, technical performance data of this herd were collected from the sow management program (Agrovision BV, The Netherlands). Technical data from the current and subsequent litters (*N* = 3,907 litters) were collected from years 2013, 2014, 2015 and 2016. Sows were selected based on partus date in between the 1^st^ of January 2013 and the 31^st^ of December 2015. As data of 2016 was already partly available, some subsequent lactation originate from 2016. Farrowing dates of the subsequent lactations were between the 29^th^ of May 2013 and the 2^nd^ of March 2016.

For the analysis of the technical performance, sows were allocated into three groups: 1) single nursing sows (SNS), which nurse ≥11 to ≤ 14 piglets during a lactation period of ≥ 24 and ≤ 30 days, 2) double nursing sows (DNS) that nurse ≥22 to ≤28 piglets during a lactation period of ≥ 24 and ≤ 30 days. All other sows, nursing less than 11 or between 15 and 21 or more than 28 piglets and sows with a lactation period < 24 days or > 30 days were allocated in group 3) “OTHERS”. The dependent variables used in this study were: 1) mortality during the current lactation, 2) interval weaning-conception for the subsequent farrowing, 3) the number of total born piglets in the next farrowing, 4) the number of live born piglets in the next farrowing. The independent variable was the categorical variable; SNS versus DNS. Data from sows in “OTHERS” were not taken into account in the comparative analyses, because this group is, by the number of piglets or number of lactation days, not meeting the inclusion criteria for being a control for the DN strategy. In addition, only sows with a parity from two to seven were included because other parities were not eligible for double nursing. Finally, lactations were only included if information on the subsequent lactation was present, since the dependent variables 2, 3 and 4 concerned the subsequent lactation.

Piglet mortality during the current lactation was established by dividing the recorded number of dead piglets allocated to the DNS or SNS during lactation by the number of piglets weaned plus the number of recorded lost piglets in the same lactation. This outcome variable was analyzed using a mixed-effects Poisson regression analysis or mixed-effects negative binomial model in case of over dispersion. A mixed-effects model was used to account for the presence of multiple lactations per sow. The number of dead piglets was the dependent variable and the number of weaned piglets plus the number of dead piglets was used as the exposure variable. All other outcome variables 2) interval weaning-conception for the subsequent farrowing, 3) the number of total born piglets in the next farrowing and 4) the number of live born piglets in the next farrowing were checked for normality. Based on a visual inspection of the normality plot, either a parametric test (mixed-effects generalized linear regression) or a non-parametric test (Wilcoxon rank-sum test; not possible to correct for multiple sow observations) was performed. For all outcome variables accounts that (when possible) a model is used in which a random sow effect is included and the effect of SN/DN is corrected for year of DN/SN, parity during DN/SN.In addition, possible effect-modification by parity is explored using the addition of an interaction term with SN/DN. A *p*-value of ≤0.05 was considered significant and a *p*-value between >0.05 and < =0.10 a trend. Analyses were performed in Stata version 14 [17].

## Results

The sows in this herd were highly prolific as 32.8 piglets were weaned per sow per year (PSY) in 2013 and 32.9 and 32.4 PSY in 2014 and 2015, respectively. The mean live born piglets (LBP) per litter were 15.06, 15.22 and 15.02 respectively in 2013, 2014 and 2015. The farrowing rate per year was: 87.6% in 2013, 90.4% in 2014 and 86.5% in 2015. Interval weaning to conception was 6.8 days in 2013, 6.9 days in 2014 and 7.8 days in 2015.

From 02-01-2013 until 31-12-2015 in total 3,907 born litters (2013 *N* = 1,268, 2014 *N* = 1,308 and 2015 *N* = 1,331) were recorded. The mean total born piglets per litter (TBP) was 16.24 with a standard deviation of 3.53 piglets and the mean live born piglets (LBP) was 15.10 ± 3.35.

**Fig. 2:**
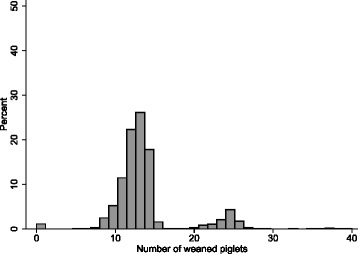
Distribution (%) of the number of piglets weaned per sow per lactation in three years (2013–2015) of all recorded nursings (*N* = 3,907)*.* In the distribution of the number of weaned piglets per sow two groups can be identified. Sows that nurse and wean one litter and DNS that nurse and wean two litters

As the selection of double nursing sows was done by the farmer based on defined sow characteristics and not by random assignment, the frequency of double nursing was not equal in all parities. In 3 years 307 out of 3,907 (=7.9%) lactations were DN lactations (Table 1). 2,841 were SN (=72.7%) and 759 nursings (=19.4%) belonged to the group “OTHERS”. DNS were mainly selected from sows of parity 2 to 5 (Table 1). DNS were frequently selected again for DN in the subsequent lactation. When a subsequent lactation was present for DNS (*N* = 250), DN was applied again in 25.2% (*N* = 63) of the sows. 57.2% was SN in the subsequent lactation (*N* = 143) and 17.6% (*N* = 44) belonged to the group “OTHERS”. In Fig. 2 the distribution of number of piglets weaned sow per lactation of all nursings in three years (2013–2015) is shown (*N* = 3,907).

**Table 1:** Distribution of the different nursing strategies per parity for all lactations during a three years period (2013–2015) (*N* = 3907)

Parity	1	2	3	4	5	6	7	8	9
	(*N* = 667)	(*N* = 594)	(*N* = 596)	(*N* = 528)	(*N* = 483)	(*N* = (421)	(*N* = 365)	(*N* = 249)	(*N* = 4)
SNS	83,7%	71,4%	73,2%	71,2%	70,8%	76,7%	70,7%	48,6%	75,0%
DNS	0,3%	10,9%	12,1%	11,9%	11,4%	6,2%	3,8%	4,0%	0,0%
OTHER	16,0%	17,7%	14,8%	16,9%	17,8%	17,1%	25,5%	47,4%	25,0%

Single nursing sows (SNS) nurse ≥11 - ≤14 piglets during a lactation period of ≥ 24 and ≤ 30 days. Double nursing sows (DNS) nurse ≥22 - ≤28 piglets during a lactation period ≥ 24 and ≤ 30 days. OTHER are sows, nursing less than 11 or between 15 and 21 or more than 28 piglets and sows with a lactation period < 24 days or > 30 days

In total, 3,148 nursings could be characterized as either SN (*N* = 2,841) or DN (*N* = 307). For 2,498 of the 3,148 nursings, information on the subsequent litter was available (2,248 for SN and 250 for DN). In 650 cases, the subsequent information was not available due to culling (*N* = 328, 302 for SN and 26 for DN), or the subsequent litter information was not available yet (*N* = 322, 291 for SN and 31 for DN). As parity-one sows were not used as DNS and the majority of eighth parity sows were culled after weaning, resulting in no subsequent litter, only performance data of sows with parity >1 and < 8 were included in the analysis. A total of 2,039 nursings were eligible for comparative analyses (1,791 SN and 248 DN). The 2,039 lactations originate from 770 sows. A single lactation (including subsequent lactation) was included for 28.2% of the sows. For 71.8% of the sows, more than one lactation was included, with a maximum of 6 lactations.

### The effect of double nursing in the current litter

As expected there was no difference in TBP and LBP in the different nursing strategies during the current lactation (Table 2). However, the DNS weaned more piglets then all other groups as this was the effect of the applied nursing strategy.

**Table 2:** Technical performance data in the current lactation of parity 2–7, per nursing strategy, in a three year period (2013–2015)

		Parity		# Total Born Piglets (TBP)		# Live Born Piglets (LBP)		Piglet mortality (%)		Lactation length (days)		# Weaned Piglets per Litter (WPL)	
	Count	Mean	Stdv	Mean	Stdv	Mean	Stdv	Mean	Stdv	Mean	Stdv	Mean	Stdv
SNS	1,791	4.11	1.64	16.68	3.44	15.60	3.23	7.13	8.06	27.08	1.31	12.64	0.98
DNS	248	3.79	1.41	16.29	3.44	15.44	3.28	5.10	4.83	27.59	1.52	24.17	1.31

Single nursing sows (SNS) nursed ≥11 - ≤14 piglets during a lactation period of ≥ 24 and ≤ 30 days. Double nursing sows (DNS) nursed ≥22 - ≤28 piglets during a lactation period ≥ 24 and ≤ 30 days

Piglet mortality in single nursing sows (*N* = 1,791) was 7.13% (95% confidence interval (CI): 6.76–7.50%) versus 5.10% (95% CI: 4.50–5.70%) in double nursing sows (*N* = 248). Since there was significant over dispersion, a negative binomial model was used to analyze the difference in mortality. The mortality during DN was significantly (*p* < 0.001) lower than during SN (incidence rate ratio (IRR) = 0.66 [95% CI: 0.57–0.76]), when corrected of year and parity (Table 3). There was no significant effect-modifying role of parity (overall *P*-value of 0.28).

**Table 3:** Multivariable model for mortality including DN/SN, year and parity with random sow effect, during the period 2013–2015 (*N* = 2,039)

Factor	Category	IRR	95% CI	Count
Nursing type	SN	reference		1,791
	DN	0.66	0.57–0.76	248
Year of weaning	2013	reference		638
	2014	1.22	1.08 –1.37	784
	2015	1.13	1.0 −1.29	617
Parity	2	reference		442
	3	0.89	0.77 – 1.02	424
	4	0.94	0.81–1.09	378
	5	0.97	0.83 –1.13	325
	6	0.83	0.70 – 0.98	283
	7	0.72	0.58–0.88	187

### The effect of double nursing in the subsequent litter

The mean interval weaning to conception (IWC) in SNS was 6.06 (95% CI: 5.76–6.35) and the mean interval weaning to conception of DNS was 5.98 (95% CI: 5.23–6.72) (Table 4). The IWC was not significantly different between DN and SN (Wilcoxson Rank sum test, *p* = 0.30).

**Table 4:** Reproductive performance data of parity 2–7 in the subsequent litter, classified by nursing type in the previous lactation in 2013–2015

		Interval Weaning to Conception (days)		# Total Born Piglets (TBP)		# Live Born Piglets (LBP)		Piglet mortality (%)		Lactation Length (days)	
	Count	Mean	Stdv	Mean	Stdv	Mean	Stdv	Mean	Stdv	Mean	Stdv
SNS	1,791	6.05	6.46	17.17	3.31	15.80	3.17	8.69	11.63	27.00	4.11
DNS	248	5.98	5.93	16.23	3.52	14.99	3.61	9.43	13.99	26.90	2.93

Single nursing sows (SNS) nursed ≥11 - ≤14 piglets during a lactation period of ≥ 24 and ≤ 30 days. Double nursing sows (DNS) nursed ≥22 - ≤28 piglets during a lactation period ≥ 24 and ≤ 30 days

A significant negative effect of DN was found on total born piglets and live born piglets in the subsequent litter (Table 5 and 6) (In both cases a mixed-effects generalized linear regression was used). DNS had significantly less TBP (−0.96 (95% CI −1.39 - -0.53) and LBP (−0.95 (95% CI −1.37 - -0.52) in the subsequent litter than the SNS, when corrected for the previous year and parity. There was no significant effect-modifying role of parity in both cases (overall *P*-value of 0.97 for TBP and 0.54 for LBP).

**Table 5:** Multivariable model for total born piglets in the next farrowing including DN/SN, year and parity with random sow effect, during the period 2013–2016 (*N* = 2,039)

Factor	Category	Coefficient	95%CI	Count
Previous nursing type	SN	reference		1,791
	DN	−0.97	−1.39 – −0.53	248
Year of previous weaning	2013	0.31	−0.07 – 0.69	638
	2014	0.43	0.11– 0.75	784
	2015	reference		617
Previous parity	2	0.11	−0.42– 0.65	442
	3	0.56	0.03 –1.09	424
	4	0.81	0.28–1.34	378
	5	0.71	0.17–1.24	325
	6	0.64	0.11–1.18	283
	7	reference		187

**Table 6:** Multivariable model for live born piglets in the next farrowing including DN/SN, year and parity with random sow effect, during the period 2013–2016 (*N* = 2,039)

Factor	Category	Coefficient	95% CI	Count
Previous nursing type	SN	reference		1,791
	DN	−0.95	−1.36–−0.52	248
Year of previous weaning	2013	0.17	−0.19–0.54	638
	2014	0.32	0.00–0.64	784
	2015	reference		617
Previous parity	2	1.12	0.60–1.65	442
	3	1.37	0.85–1.89	424
	4	1.23	0.70–1.75	378
	5	0.96	0.43–1.49	325
	6	0.80	0.26–1.33	283
	7	reference		187

## Discussion

By implementing double nursing as a management strategy, all suckling piglets had daily access to sow milk and none of the piglets had to be weaned early and/or reared solely on formula. This case report shows that DN can be applied, taking into account the selection criteria for sows, ensuring a nursing period of four weeks for all piglets. Unfortunately, not all selection criteria could be incorporated in the multivariable models resulting in a less clear estimate of the true effect of SN/DN. We expect that the effects of SN/DN that were found, are somewhat influenced by effect of selection criteria like sow characteristics. The positive effect of double nursing on piglet mortality in the current lactation on the presented farm is very encouraging, but mortality in DNS can be somewhat biased by data recording by the herdsman and (above mentioned) selection criteria for DN and SN-piglets. Firstly, piglet mortality is not recorded pre- and post-litter-allocation separately. Thus, piglet mortality of the DNS, misses the mortality of non-viable piglets in the litter of “donor” sow in hours before litter allocation. This data recording bias results in a lower calculated mortality in DNS. Secondly, piglets and sows are not randomly selected for DNS, but based on piglet viability and sow motherly behavior. This selection bias has likely contributed to positive effect of DN on piglet mortality that was found. Unknown is the additional effect of the implementation of the double nursing strategy on the overall herd piglet mortality as this study only consists of the recordings of one herd.

Due to milk production almost all sows lose bodyweight during the lactation period [18]. Excessive body condition loss during lactation may have a negative effect on the weaning to estrus interval, the weaning to conception interval and the litter size of the subsequent litter [18–22]. The technical performance data of the herd of this study showed a negative effect of DNS on the litter size of the subsequent litter (SNS: 17.17 95% CI 17.02–17.32 versus DNS: 16.23 95% CI 15.79–16.67). Total milk yield per day depends on the number of successful nursings multiplied by the number of producing teats [7, 16, 23, 24]. In contrast to the first days post-partum, where sows initiate 80–100% of nursings, during the second part of the lactation period piglets more and more initiate nursings by stimulating the sow. Thus, nursing intervals can be driven by piglet behavior [25, 26]. In DNS due to piglet behavior, we assume the absolute number of successful nursings to be higher and in that way be an explanation of a higher loss of body condition [16, 22]. It has been shown that body condition losses of more than 10% in parity 2 can result to lower subsequent litter size and increased weaning to estrus interval [18]. Because body weights, body condition scores nor back fat at farrowing or weaning were scored, we can only assume that body condition loss during lactation is the reason for the effect in the parity 2 sows. Based on the shown result more focus on body condition loss and high feed intake is necessary for the future.

For this herd instead of weaning to estrus, weaning to conception interval was analyzed. Our data show no effect of DNS on the interval weaning to conception (*p* = 0.30) in contrast to a previous publication based on 20 Danish sow herds [11]. Nurse sows were defined as sows weaning their own litter at least 18 days postpartum and thereafter nursing another litter (nurse litter) before service. Foster sows had a longer lactation length and an increased weaning to estrus interval with no effect on subsequent farrowing rate, but a positive effect on the subsequent litter size. DNS was not associated with extra culling of sows as 26 of 307 (=8.5%) of DNS were culled versus 302 of 2,841 (=10.6%) SNS. Whether the negative effect on litter size in double nursing is compensated by absence of a negative effect on the cycle length, compared to the nurse litter strategy, is still unknown as we did not compare the nurse litter strategy and the double nursing strategy within this herd at the same time.

## Conclusion

In this herd the DN appeared to be a successful strategy to nurse the number of piglets born in one-week-farrowing batch by the sows farrowing within the same batch. DNS seemed to experience a lower piglet mortality, despite the double nursing strategy, even though some methodological issues withhold a firm statistical conclusion in this case. Still, no relevant harmful effects were found, despite a slight negative effect on litter size in subsequent litters. DNS might be a good alternative to other fostering strategies. Future research should be based on multiple herds and include effects of DNS on litter growth and body condition of the sows The authors want to articulate that the effects of DN that were found in this study only apply when sows meet the specific criteria, as random attribution of SN/DN was not performed.

## Abbreviations

CI: Confidence interval
DN: Double nursing
DNS: Double nursing sow(s)
IWC: Interval weaning conception
IRR: Incidence Rate Ratio
LBP: Live born piglets
PSY: Piglet per sow per year
SN: Single nursing
SNS: Single nursing sow(s)
Stdv: Standard deviation
TBP: Total born piglets
WPL: Weaned piglets per litter
